# Multiplicity and Diversity of *Plasmodium vivax* Infections in a Highly Endemic Region in Papua New Guinea

**DOI:** 10.1371/journal.pntd.0001424

**Published:** 2011-12-20

**Authors:** Cristian Koepfli, Amanda Ross, Benson Kiniboro, Thomas A. Smith, Peter A. Zimmerman, Peter Siba, Ivo Mueller, Ingrid Felger

**Affiliations:** 1 Swiss Tropical and Public Health Institute, Basel, Switzerland; 2 University of Basel, Basel, Switzerland; 3 PNG Institute of Medical Research, Goroka, Papua New Guinea; 4 Centre for Global Health and Disease, Case Western Reserve University, Cleveland, Ohio, United States of America; 5 Walter and Eliza Hall Institute, Parkville, Victoria, Australia; 6 Barcelona Centre for International Health Research, Barcelona, Spain; New York University, United States of America

## Abstract

*Plasmodium vivax* is highly endemic in the lowlands of Papua New Guinea and accounts for a large proportion of the malaria cases in children less than 5 years of age. We collected 2117 blood samples at 2-monthly intervals from a cohort of 268 children aged 1 to 4.5 years and estimated the diversity and multiplicity of *P. vivax* infection. All *P. vivax* clones were genotyped using the merozoite surface protein 1 F3 fragment (*msp1*F3) and the microsatellite MS16 as molecular markers. High diversity was observed with *msp1*F3 (*H*_E_ = 88.1%) and MS16 (*H*_E_ = 97.8%). Of the 1162 *P. vivax* positive samples, 74% harbored multi-clone infections with a mean multiplicity of 2.7 (IQR = 1–3). The multiplicity of *P. vivax* infection increased slightly with age (*P* = 0.02), with the strongest increase in very young children. Intensified efforts to control malaria can benefit from knowledge of the diversity and MOI both for assessing the endemic situation and monitoring the effects of interventions.

## Author Summary

The parasite *Plasmodium vivax* is the second most frequent cause of malaria in humans. In the Maprik area in lowland Papua New Guinea, *P. vivax* and *P. falciparum* are sympatric each with a prevalence of around 50%. Longitudinal samples from 268 children aged 1 to 4.5 years over 16 months were collected. The 1162 blood samples positive for *P. vivax* were genotyped for two size-polymorphic molecular markers. A very high parasite diversity was observed. The number of co-infecting parasite clones per carrier (multiplicity) was nearly twice as high for *P. vivax* as for *P. falciparum* despite the similar prevalences of the species. The *P. vivax* multiplicity increased with age, with the strongest increase in young children below 1.5. This is likely to be a consequence of fast acquisition of immunity against *P. vivax* malaria and also of relapses, the release of long-lasting, silent liver stages to the blood stream. This is the first dataset from a highly endemic setting that presents data on a large number of individual *P. vivax* clones genotyped with highly diverse markers.

## Introduction

Malaria caused by *Plasmodium vivax* infection is increasingly recognized as a public health burden. The worldwide population at risk is estimated to be 2.85 billion, with high prevalences observed in locations throughout Southeast Asia and the Pacific [1]. Even though *P. vivax* epidemiology is less well studied and understood compared to that of *P. falciparum*, it is thought that *P. vivax* will present a greater challenge on the way to elimination of malaria outside Africa. Papua New Guinea (PNG) presents a variety of different climatic and ecological zones which have differing levels of malaria transmission [2] with a high burden of *P. vivax* in the tropical lowlands. The occurrence of high prevalence and morbidity marks locations in PNG as suitable field sites for *P. vivax* drug trials [3], [4] and potentially also for future vaccine trials [5].

In Maprik in northern PNG, both *P. vivax* and *P. falciparum* are highly prevalent [6]. The incidence of *P. vivax* clinical episodes has been shown to peak in the second year of life, while that of *P. falciparum* increases until the fourth year [7], [8]. Children between one and five years are considered to be a target age-group for *P. vivax* vaccine trials [5]. While previous studies have provided baseline data on clinical incidence rates, epidemiological patterns and the age-distribution of disease [8], [9], genotyping data on individual *P. vivax* clones describing their diversity and molecular epidemiological parameters are scarce. For *P. falciparum*, the mean number of concurrent infections per patient (multiplicity of infection, MOI) has been used as one of several measures of the impact of interventions. MOI is crucial to assess the risk that an individual carries a drug resistant parasite and to evaluate levels of inbreeding [10].

Here we describe the genetic diversity and multiplicity of infection of a *P. vivax* population in an area of high malaria prevalence in the Maprik District in PNG. Data were obtained from children aged 1 to 4.5 years who were followed-up over 16 months [8]. We make use of two markers to genotype individual *P. vivax* clones, one a microsatellite and the other a region of the *msp1* gene, encoding the Merozoite Surface Protein 1. While the microsatellite MS16 (located on NCBI contig XM_001615468.1) is considered a neutral marker that has been used in a number of population genetics studies [11], [12], *msp1* (XM_001614792) encodes a potential vaccine candidate (reviewed in [13]) and its diversity has been studied in different settings [14], . In a previous study, the two markers showed robust PCR amplification and high diversity with a risk of less than 1% that two clones share the same two-loci haplotype [17].

## Methods

### Ethics statement

The cohort study was approved by institutional review boards of the PNG Medical Research Advisory Committee (approvals 05.19 and 09.24), University Hospitals Case Medical Center (Cleveland, Ohio USA), and the Ethikkommission beider Basel (approval 03/06). Informed written consent was provided by the parents or legal guardians of each child.

### Study site and design

The cohort study was conducted in the Ilaita area, Maprik District, East Sepik Province, PNG between April 2006 and August 2007. The study area has hyper- to holoendemic perennial transmission with moderate seasonal variation [8], [18], [19], [20]. *P. vivax* infections are the most prevalent infection in young children and remain frequent into adulthood, while *P. falciparum* is the predominant infection in children over 4 years of age [6], [21].

268 children aged 1 to 3 years at enrolment were followed-up over a period of 16 months. As part of the cohort study, the children were visited every two months with blood samples taken at least for one and, for some surveys, on two consecutive days. In the analysis presented here, only blood samples taken on the first day were included. The prevalence of Plasmodium species by microscopy in the study population was 44.3% for *P. vivax*, 32.6% for *P. falciparum* and 4.2% for *P. malariae* [8]. Defining clinical episodes as the presence of fever >37.5°C (axillary temperature measured twice and a third time if the difference was above 0.3°) or history of fever during the last 48 hours together with parasitaemia observed by light microscopy, the clinical incidence rates were 2.56 *P. falciparum* and 2.46 *P. vivax* episodes per child per year [8]. Children presenting with parasitologically confirmed malaria (i.e. positive blood slide or RDT) were treated with Coartem. Details of the study have been published previously [8], as well as genotyping data of the population of *P. falciparum* clones in this cohort [22].

### Species detection, genotyping and data analysis

Finger prick and venous blood samples were collected and DNA was extracted as previously described [8]. The presence of *P. falciparum*, *P. vivax*, *P. ovale* and *P. malariae* was detected by light microscopy as well as by post-PCR Ligase Detection Reaction (LDR) [8], a molecular method for the detection and species identification of malaria parasites [23]. All samples which were *P. vivax* positive by light microscopy or LDR plus 88 negative baseline samples were selected for genotyping.

The selection of highly diverse molecular markers is crucial for assessing MOI in molecular epidemiology studies. Based on our previous results [17], we selected the polymorphic marker gene *msp1*F3 and the microsatellite MS16 for genotyping. In contrast to population genetic studies, where large numbers of neutral markers of moderate to high diversity (e.g. microsatellites) are generally analyzed, a small number of highly polymorphic markers are ideal for tracking clones in epidemiological studies for two reasons Where concurrent infections with several clones are common construction of haplotypes combining data from several PCR amplified markers is difficult. In addtion, when MOI is defined as the maximum number of clones by any of the markers typed, the risk of overestimating MOI due to PCR artefacts increases with the number of markers analyzed, in particular for microsatellite amplification where PCR artefacts caused by polymerase slippage are of concern [24] (Tables A and B in Text S1). Any highly polymorphic marker is suitable for studying the epidemiology of multiple infections. As long as high diversity is maintained, there is no need for selective neutrality.

In this molecular epidemiological study, the coding sequence *msp1*F3 was chosen because it harbours a more complex repeat structure than microsatellites. This has the advantage that PCR artefacts due to slippage are rare for *msp*1F3. Our second marker was MS16, a highly polymorphic microsatellite that lacks dominant alleles.

PCR and capillary electrophoresis were performed with slight modifications of the published protocol [17] to save costs and labour time: a multiplex primary PCR was done with the primers for the 2 markers *msp1*F3 and MS16 followed by individual nested PCRs for *msp1*F3 and MS16. The primary PCR was done in a volume of 20 µl containing 1 µl template DNA, 0.25 µM of each primer (Eurofins MWG Operon), 0.3 mM dNTPs (Solis BioDyne), 2 mM MgCl_2_, 2 µl Buffer B (Solis BioDyne) and 5 U *Taq*FIREPol (Solis BioDyne). As we expected low parasitemia in samples negative by microscopy, 2 µl DNA instead of 1 µl were used for the primary PCR. 1 µl primary PCR product was used as the template for the nested PCR, which was performed in a volume of 20 µl containing 0.25 µM of each primer (Applied Biosystems), 0.2 mM dNTPs (Solis BioDyne), 2 mM MgCl_2_, 2 µl Buffer B (Solis BioDyne) and 1.5 U *Taq*FIREPol (Solis BioDyne). The forward primers for the nested PCR were labelled with fluorescent dyes: 6-FAM for *msp1*F3, NED for MS16. Cycling conditions were as follows: initial denaturation 95°C for 1 minute, then 30 cycles (primary PCR) or 25 cycles (nested PCR) with 15 seconds denaturation at 95°C, 30 seconds annealing at 59°C and 30 seconds elongation at 72°C plus a final elongation of 5 minutes at 72°C. Subsequently, capillary electrophoresis was performed as described [17].

The PCR data was analysed using the GeneMarker® programme version 1.85 (SoftGenetics). Based on experience from preliminary studies, peaks above a cut off of 1000 units relative fluorescent intensity (RFU) were considered true amplification products, all peaks below this cut off were considered background noise as well as lesser peaks in the vicinity of strong peaks reaching 40% (*msp1*F3) and 70% (MS16) of their height. Occasionally the fluorescence intensity differed between plates or samples as indicated by varying signal intensities of the commercial size standard. To compensate for this technical shortfall, the standard cut off value was lowered from 1000 to 300 RFU if signal intensities of both sample peaks and size standard peaks were low (generally below 1000 RFU). This practice was justified by a greater agreement in positivity at both loci. As a consequence, the proportion of samples positive only for a single marker dropped from 12% to 10%. All samples were checked visually (after blinding of samples) for stutter peaks thereby excluding one *msp1*F3 and 25 MS16 samples from further analyses.

The genotyping method was validated by typing a subset of 28 samples for both markers in duplicate. 80% of *msp1*F3 clones and 88% of MS16 clones were detected in both replicates (Tables C and D in Text S1). An important reason for the imperfect detection of clones is the low concentration of template DNA in samples with scanty parasitemia, where partial amplification of all templates seems to be governed by chance. In serial dilutions of DNA in field samples, we have demonstrated this effect by performing PCR amplification in triplicate for each dilution (Tables E and F and Figures A and B in Text S1). At low DNA concentrations, the allelic composition of a blood sample differed between replicates with individual clones detected in an apparently random fashion (out of several clones detected in undiluted DNA).

### Data analysis

Alleles were grouped into bins of 3 base pairs, defined by the expected size differences in the two markers: 3 base pairs (bp) for the coding region of Pv*msp1* as well as for microsatellite MS16 harbouring a 3 bp repeat unit. When a single genotype was observed with both markers, a blood sample was defined as single clone infection. In multiple clone infections, the highest number of clones observed for either marker defined the combined MOI of a blood sample. We used the kappa statistic to describe agreement between the molecular markers after correcting for chance agreement.

The determination of MOI for single and double clone infections was validated by genotyping a subset of samples with 12 additional markers. MOI = 1 was confirmed in 67/92 (72.8%) samples and MOI = 2 in 31/32 (96.9%) samples (Tables A and B in Text S1).

Although the distributions of MOI are skewed, we present the mean MOI, a common measure, to allow comparisons with other studies. We estimated the effect of age (at the time of the survey in 6 months age groups) and season on prevalence and MOI using regression models. To account for multiple visits per child, we included a random effect for child. The models were implemented in STATA version 10 [25] and WinBUGS version 1.4 [26].

The genetic diversity of a given locus in a population is expressed by the virtual heterozygosity *H*_E_, i.e. the probability that two clones taken at random from the population carry different alleles. *H*_E_ was calculated using the formula
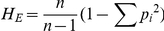
where *n* is the number of clones analysed and *p* is the frequency of allele *i*. *H*_E_ of *msp1*F3 and MS16 were determined by using only the first *P. vivax* positive sample of each study participant; *H*_E_ of *msp1*F3/MS16 haplotypes by using the first single clone infection per patient. This procedure prevents potential sampling bias due to repetition of persisting clones from the same individual. Linkage between markers was assessed using LIAN 3.5 [27]. Linkage disequilibrium measured from only two markers cannot provide information on inbreeding and was used only to provide evidence that the markers occur independently of each other.

## Results

Of the 2117 blood samples collected in cross sectional surveys, 1340 were genotyped since they were positive by microscopy or LDR. Of the 88 microscopy and LDR negative samples that were selected for genotyping, only 2 (2.3%) were positive. This low proportion did not justify genotyping all negative samples.

Nested PCR for the two *P. vivax* genotyping markers provided an amplification product from at least one marker from 1162 samples. *msp1*F3 PCR products were obtained from 1094 samples MS16 PCR products from 1118 (Table 1). In 1050 samples (90.3%), positive results were obtained for both markers. The two markers agreed well on *P. vivax* positivity (kappa = 0.71), although the difference in PCR efficiency (McNemar's test: *P* = 0.026) suggests a slightly higher sensitivity of the MS16 PCR.

**Table 1:** Diversity and multiplicity of infection of *P. vivax* in Papua New Guinea.

	No. of positive samples	No. of clones	Multiplicity of infection	Allelic richness	Virtual Heterozygosity (*H*_E_)
*msp1*F3	1094 (79)*	2480 (173)*	2.27	57 (31)*	0.881 (0.874)*
MS16	1118 (80)*	2542 (175)*	2.27	103 (65)*	0.976 (0.977)*
combined	1162	NA**	2.69	NA**	NA**
Single clone infections	219 (25)*	219 (25)*	1	154 (25)*	0.991 (0.96)*

*In brackets numbers for baseline only.

**NA = Not applicable.

### Prevalence

The overall prevalence of *P. vivax* based on positivity by PCR was 55%. The prevalence was lowest in children under 1.5 years at 44% and reached 62% in children aged 3 to 3.5 years. The increase of prevalence by age at the time of the survey was significant (*P* = 0.005) largely driven by the lower prevalence in children less than 1.5 years. Without this youngest age group no evidence of a trend was observed. No major seasonal trend in *P. vivax* prevalence was observed with the exception of a slight peak in September (*P* = 0.17).

### Allelic diversity

**Figure 1:**
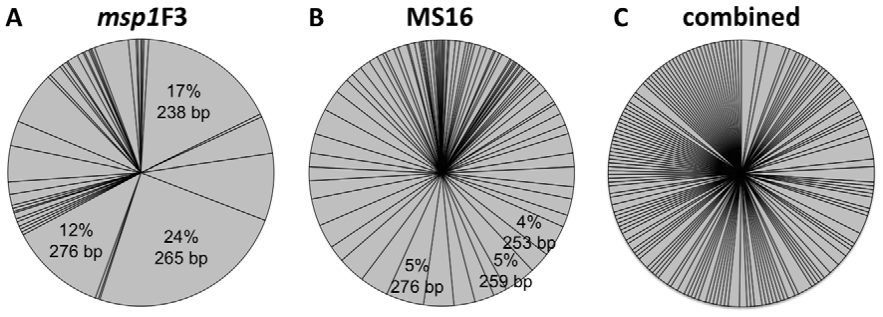
Allelic frequencies of *P. vivax* genotyping markers. Allelic frequencies of markers *msp1*F3 (A) and MS16 (B) and the combined *msp1*F3-MS16 haplotypes (C). For *msp1*F3 and MS16 the frequencies of the 3 most frequent alleles and the respective sizes of the amplified product are given.

In 1162 samples positive for *P. vivax*, 57 different *msp1*F3 and 103 different MS16 alleles were detected (Figures 1A and 1B). Virtual heterozygocity *H*_E_ was 97.8% for MS16, 88.1% for *msp1*F3 and 99.1% for *msp1*F3-MS16 haplotypes determined in single-clone infections (Table 1). In 219 single clone infections from 148 patients, a total of 154 different haplotypes were observed with the most common haplotype detected in only six individuals (Figure 1C). We tested this data set for independence of the two molecular markers. No linkage disequilibrium was observed (*I_A_^S^* = −0.0001, *P* = 0.53).

### Multiplicity of infection

**Figure 2:**
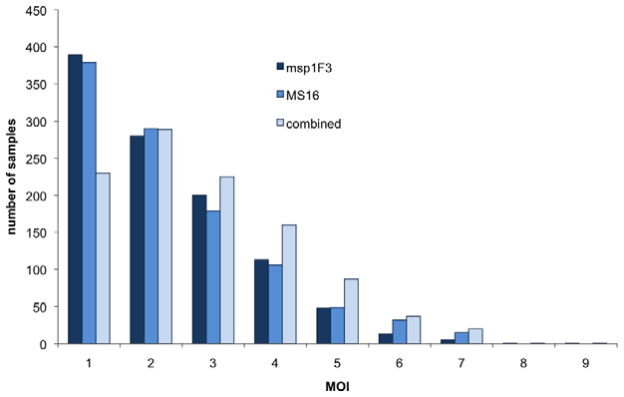
Distribution of multiple clone infections. Distribution of multiplicity of infection as detected by the markers *msp1*F3 and MS16 as well as both markers combined. Only samples with positive results for both markers were included (n = 1050).

The MOI was determined for each marker separately, as well as for both markers combined. In *P. vivax* positive samples, the mean MOI was 2.27 for each marker individually and 2.69 when calculated from the maximal number of clones per sample by any marker. Among samples for which positive results were obtained from both markers, MOI was concordant in 38% (397/1050) (kappa = 0.17). A difference of one clone was observed in 38.5% (404/1050) of samples. The frequency distribution of MOI plotted separately for *msp1*F3 and MS16 was compared to the combined MOI (Figure 2). A single marker slightly underestimated MOI. Multiple clone infections were observed in 63% of all positive samples by *msp1*F3 and in 61% by MS16. When results of both markers were combined, the proportion of multiple clone infections increased to 74% (Table S1). Likewise, the proportion of samples with a MOI of 3 or higher was underestimated based on a single marker. Of the 531 samples with a combined MOI between 3 and 9, the combined MOI result was reproduced only in 279 samples (52%) by *msp1*F3 alone and in 324 samples (61%) by MS16 alone.

**Figure 3:**
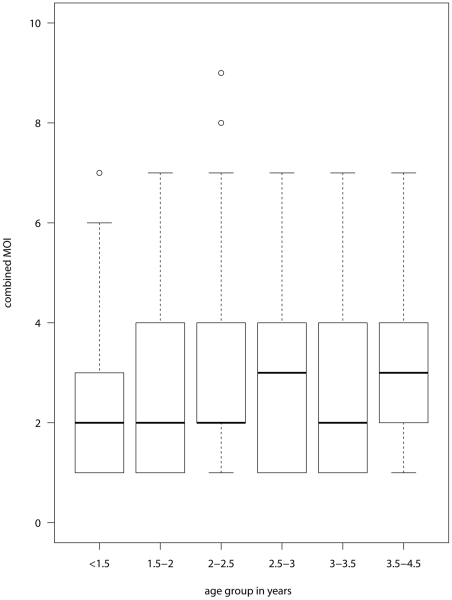
Boxplot of MOI by age group. The median is represented by the central line. The box represents the interquartile range from the 25^th^ to 75^th^ centiles. The whiskers extend to the most extreme data point which is no more than 1.5 times the interquartile range from the box, points beyond this are plotted individually.

**Figure 4:**
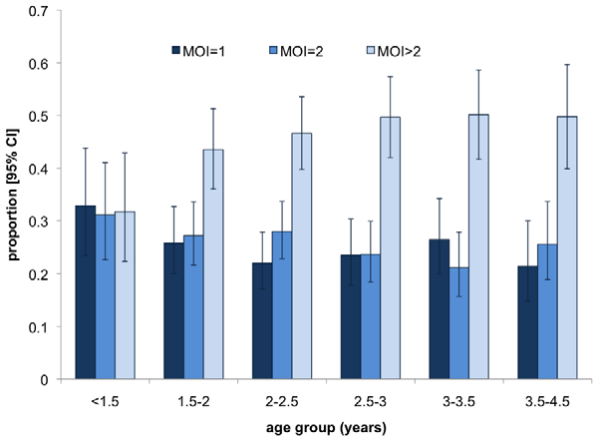
Proportions of multiple clone infections by age group. Proportion of children with *P. vivax* multiplicity of infection of 1, 2 and 3 to 9 by age. Results from two markers combined are shown. Error bars show 95% confidence intervals.

The mean MOI of *P. vivax* was associated with age. In children up to 1.5 years the mean MOI was 2.4 increasing slightly up to 2.8 in children 3.5 to 4.5 years of age (Figure 3, *P* = 0.02). If the youngest children below 1.5 years were excluded, no significant trend was observed (*P* = 0.23). The increase of the proportion of children bearing more than two clones was more pronounced (Figure 4). In the 18 youngest children aged 300 to 400 days, we observed a low MOI of 1.67 and only two children (11%) carried more than 2 clones. There was no significant seasonal variation in MOI (*P* = 0.50).

## Discussion

We have genotyped *P. vivax* parasites in a cohort of 268 children from an area of Papua New Guinea with sympatric *P. falciparum* and *P. vivax* with prevalences of 49.6% and 53.0% respectively in this cohort at enrolment [8]. The ecology and epidemiology of *P. vivax* differs from that of *P. falciparum* in several aspects. Parasite densities are generally lower, which is likely to affect prevalence data generated by both microscopically or molecular diagnosis [28], [29]. Gametocytes appear shortly after an infection is established in a host [30], with implications for transmission and the frequency of sexual recombination, and the occurrence of relapses leads to appearance of new genotypes in the blood stream independent of mosquito transmission.

Our genotyping enables the distinction of individual parasites within the human host and thus the assessment of MOI. The mean MOI for *P. vivax* in our cohort was 2.7 and 73.6% of samples carried multiple clones (combined results from two independent markers). This compares to a substantially lower *P. falciparum* MOI of 1.5 in the same cohort determined by the marker *msp2* and only 35.2% of samples carrying multiple clone infections [31].

The multiplicity of *Plasmodium* infections depends on a number of factors including transmission intensity or the duration of infection as the result of loss of infection and antimalarial treatment. An additional factor, unique to *P. vivax*, contributes to the number of blood stage infections circulating in the blood: relapses of semi dormant liver stages. Previous studies have shown that relapses often genetically differ from already present blood stage parasites [32], [33] and thus lead to increased MOI. As Coartem does not clear liver stage parasites and the level of treatment in our cohort was high, the combined effect of treatment and relapses is likely to enhance differences between *P. vivax* and *P. falciparum* MOI. In addition, as mosquitoes biting people harbouring multi-clone infections are more likely to transmit several clones concurrently [34], [35], the higher MOI among *P. vivax* blood-stage parasites will increase the likelihood that multi-clone *P. vivax* infections are transmitted in a single mosquito bite.

Under intense transmission such as found in lowland PNG, the MOI of *P. vivax* species increases with age, with the increase most pronounced in children below 1.5 years. In an earlier study, no evidence of differences between children aged 4 to 14 were found [36]. This increase in early childhood may at least in part be related to the increased exposed body surface with child growth thus leading to higher rates of mosquito bites and consequently risk of infection [37]. In addition, with rapidly increasing immunity fewer *P. vivax* infections may reach high densities which are associated with febrile illness and antimalarial treatment [8] and the average duration of a *P. vivax* infection may increase with age.

Both the mean MOI and prevalence using genotyping data showed no pronounced seasonality, concurring with previous findings using light microscopy and LDR detection [8]. In contrast, the incidence of clinical disease increased in the wet season [8]. The lack of annual fluctuations in *P. vivax* prevalence and MOI observed in PNG is likely to be caused by relapses during periods where there is less mosquito transmission.

The proportion of multiple-clone infections clearly differs from observations from countries of lower *P. vivax* endemicity. We observed polyclonal infections in three out of four samples. Even if numbers cannot be compared directly to other studies using other genotyping protocols (higher numbers of markers increase the chance of observing several clones at least in one marker), this proportion only reaches 11 to 49% in the Amazon [12], [38], 55% in Sri Lanka [11] but 73% in Myanmar [11].

The molecular markers *msp1*F3 and MS16 showed a high degree of genetic diversity. While three *msp1*F3 alleles reached frequencies above 10%, the distribution of MS16 alleles is more homogenous with the highest frequency of 5%. The selected markers are suitable for studies where high resolution discrimination between *P. vivax* clones is required, such as longitudinal tracking of clones or discrimination between existing and incoming infections. The probability of two individual clones sharing the same 2-loci haplotype was below 1%. Analysing additional polymorphic markers would lead only to a minimal improvement of discrimination. As the mean MOI increases, the chance that two clones within a host share the same haplotype increases. Simulations indicate that mean MOI would be unlikely to be substantially underestimated with either marker, unless the mean MOI was greater than 6 (Amanda Ross, manuscript in preparation). The diversity observed in *P. vivax* compares well to the genetic diversity of two *P. falciparum* markers, *msp1* and *msp2*, previously determined in the same cohort [22]. MS16 was highly diverse in our cohort, and has been shown to be almost as diverse in countries of lower *P. vivax* endemicity such as Peru [12] and Vietnam [39] but was lower in Sri Lanka and Ethiopia [11].

In more than half of the samples, the number of clones detected was discordant for the two markers. Three factors contribute to such discrepancies, namely (i) differences in the discrimination power of the two markers, (ii) imperfect detection of clones in samples with low parasite densities and (iii) mutation of one of the markers occurring within a host (Text S1). Given the high diversity of both markers, we expect that limited discrimination power only accounts for a small fraction of observed discrepancies. More likely, low parasite densities around the detection limit will cause imperfect detection of clones. In a previously published analysis of clone detectability in the same set of samples, we have determined the contribution of an additional blood sample collected 24 hours later from the same children. This analysis showed that detection of genotypes by PCR is equally imperfect for both species, *P. falciparum* and *P. vivax*. Overall, 17 to 31% of all clones were missed on a single day, and detection of clones was imperfect especially in samples harboring a high number of concurrent clones [31]. In addition, in serial dilutions of parasite DNA from field samples we now show that at very low concentrations, MOI and allelic composition differed between replicates. In particular minority clones were lost. It is therefore very likely that such stochastic amplification of genotypes also occurred in our samples.

Due to its antigenicity and surface exposed location, MSP1 is considered a candidate for a malaria vaccine (reviewed in [13]). We identified 57 MSP1F3 alleles with the 3 most abundant alleles adding up to a frequency of 53%. The predominant alleles maintained stable allelic frequencies throughout the study. In a similar number of Papua New Guinean children, 27 haplotypes were observed for another potential vaccine candidate, the Duffy Binding Protein II (DBPII, XM_001615397). The 3 most frequent DBPII alleles were present in 57% of infections [40]. These results indicate high diversity of *P. vivax* antigens in PNG. With respect to vaccine development based on PvMSP1, the allelic frequencies generated in our genotyping study provide useful information on genetic diversity of this antigen.

In summary, this study provides one of the first large data sets of *P. vivax* genotypes from a highly endemic area. Our high resolution typing technique accurately determined allelic frequencies and clone multiplicity. This adds to the knowledge about *P. vivax* epidemiology and may serve as reference data for high endemicity *P. vivax* populations. The molecular parameters established could be utilized as one of several measures for effective monitoring of intervention and control of *P. vivax*, for surveillance and to parameterize mathematical models of transmission dynamics [40].

## Supporting Information

Table S1 **Genotyping results from 1162** ***P. vivax*** **positive field samples using the markers** ***msp1*** **F3 and MS16.** This table contains the number of samples for MOI = 1 to MOI = 9 for each marker separately and for the combination of both markers.
(PDF)

Text S1 **Validation of** ***Plasmodium vivax*** **genotyping based on** ***msp1*** **F3 and MS16 as molecular markers.** This text contains results from confirmation of multiplicity of infection in field samples by genotyping additional molecular markers, congruence of samples typed in duplicate, and detection of clones in serial dilutions of DNA. This file contains Tables A–F and Figures A and B.
(PDF)

Checklist S1 **STROBE checklist.**
(DOC)
