# Supplementary material for: Multiplicity and Diversity of Plasmodium vivax Infections in a Highly Endemic Region in Papua New Guinea
Source: PLoS Negl Trop Dis. 2011 Dec 20;5(12):e1424. doi: 10.1371/journal.pntd.0001424 (PMC3243695; doi:10.1371/journal.pntd.0001424)
Supplement: Table S1 — Genotyping results from 1162 P. vivax positive field samples using the markers msp1 F3 and MS16. This table contains the number of samples for MOI = 1 to MOI = 9 for each marker separately and for the combination of both markers. (PDF) [file pntd.0001424.s001.pdf]

Table S1: Genotyping results from 1162 *P. vivax* positive field samples using the markers *msp1*F3 and MS16.

	MOI as detemined by *msp1*F3	MOI as detemined by MS16	MOI combined from both markers	MOI identical by both markers
MOI=1	404	439	305	230
MOI=2	291	295	305	95
MOI=3	208	181	235	50
MOI=4	118	107	166	18
MOI=5	50	49	89	0
MOI=6	16	32	40	4
MOI=7	5	15	20	0
MOI=8	1	0	1	0
MOI=9	1	0	1	0
Sample negative for one marker and positive for the other marker	67	19	0	
Sample excluded because of PCR artifacts	1	25	0	
Total number of samples	1162	1162	1162	397
Total number of multiclonal infections	690 (63.1%)**	679 (60.7%)**	857 (73.6%)**	
Total number of single clone infections	404 (36.9%)**	439 (39.3%)**	305 (26.2%)**	230

* If values from both markers were discrepant, the higher value was accepted

** Not taking into account samples negative for this marker and samples excluded because of PCR artifacts
