# Supplementary material for: Multiplicity and Diversity of Plasmodium vivax Infections in a Highly Endemic Region in Papua New Guinea
Source: PLoS Negl Trop Dis. 2011 Dec 20;5(12):e1424. doi: 10.1371/journal.pntd.0001424 (PMC3243695; doi:10.1371/journal.pntd.0001424)
Supplement: Text S1 — Validation of Plasmodium vivax genotyping based on msp1 F3 and MS16 as molecular markers. This text contains results from confirmation of multiplicity of infection in field samples by genotyping additional molecular markers, congruence of samples typed in duplicate, and detection of clones in serial dilutions of DNA. This file contains Tables A–F and Figures A and B. (PDF) [file pntd.0001424.s002.pdf]

Text S1: Validation of *Plasmodium vivax* genotyping based on *msp1*F3 and MS16 as molecular markers

1. Confirmation of multiplicity of infection in field samples from Papua New Guinea by genotyping additional molecular markers

92 single clone infections and 32 double clone infections (based on combined results from *msp1*F3 and MS16) were typed with 12 additional markers: MS1, MS2, MS4, MS5, MS6, MS7, MS8, MS9, MS10, MS12, MS15, MS20 [1] (manuscript in preparation).

Assessing multiplicity of infection (MOI) based on a large number of markers increases the risk that MOI is overestimated because of potential within-host clonal variation [2] or stutter artefacts occurring in one marker. Intra-host clonal variation is thought to be the result of strand-slippage in a repetitive sequence, such as a microsatellite, during mitotic DNA replication. Stutter peaks are PCR artefacts observed especially when microsatellites with a simple repeat structure are amplified [3]. Despite visual checking of electropherogrammes for stutter peaks and excluding them, some PCR artifacts may be overlooked. Both processes lead to higher MOI at one locus, while the other loci remain unchanged.

To compensate for within-host clonal variation and PCR stutter peaks, we set a cut-off as follows: a sample was accepted as multi-clone infection if at least 3 loci indicated a MOI>1. Applying this cut-off, MOI=1 (based on *msp1*F3/MS16 typing) was confirmed in 67/92 samples (72.8%) (Table S1).

Table A: Confirmation of single clone infections (MOI=1) by genotyping 12 additional markers

Total no. of samples	all markers MOI=1	1 marker with MOI>1	2 markers with MOI>1	3 markers with MOI>1	>3 markers with MOI>1
92	31 (33.7%)	23 (25.0%)	13 (14.1%)	18 (19.6%)	7 (7.6%)

In sample sets typed with many markers it has been frequently observed that the highest MOI detected by any marker is not supported by other markers [4,5], suggesting occurrence of intra-host clonal variation and PCR stutter peaks. Their frequency is not known. However we can estimate it from our data in table S1 that includes results from 1288 individual PCRs (92 samples typed with *msp1*F3, MS16 and 12 microsatellites). In a total of 49 PCRs additional alleles were detected (23 samples with 1 PCR indicating MOI >1 and 13 samples with 2 PCRs indicating MOI > 1). Based on these values, we estimate the frequency of PCR results that overestimate MOI to be 49/1288 = 3.8%. The following calculation illustrates the increased risk for overestimating MOI when many markers are analyzed: assuming a 4% chance of a false-positive peak for a single marker, 96% of all samples will reveal the true MOI for this marker. However, with 14 independent markers, the chance of observing the true MOI drops to 56%.

In addition to single clone infections, we have genotyped 32 samples with MOI=2 revealed by *msp1*F3/MS16 typing for 12 additional markers. Results are given in table S2. Applying the same cut off as above (maximal two markers with higher MOI), MOI=2 was confirmed in 31/32 (96.9%) of samples.

For 4/32 (12.5%) samples, MOI=2 was not confirmed by any additional marker. We cannot rule out that these samples are in fact single clone infections with an incorrect result for either *msp1*F3 or MS16.

Table B: Confirmation of MOI=2 by genotyping 12 additional markers

Total no. of samples	all markers MOI=1 or 2	1 marker with MOI>2	2 markers with MOI>2	3 markers with MOI>2	>3 marker with MOI>2
32	20 (62.5%)	9 (28.1%)	2 (6.2%)	1 (3.1%)	0

Conclusion: When choosing the number of markers to be genotyped, there is a trade-off between number of artefacts, which increases with each additional marker, and limited resolution of multiple infections when there are high frequencies of some alleles. Using the 12 additional markers, we reproduced the *msp1*F3/MS16 genotyping results for a MOI of 1 or 2 in a reasonable proportion of the samples.

2. Congruence of samples typed in duplicate

We genotyped 28 samples in duplicate for both markers *msp1*F3 and MS16. Congruence in MOI results and allelic composition in each sample was assessed.

MOI was identical in 50% (*msp1*F3) and 71% (MS16) of duplicates (Table S3). The agreement, estimated using kappa (Table S3), was fair to high.

Table C: Congruence in MOI after genotyping 28 samples in duplicate

Difference in MOI	msp1F3	MS16	Combined results
0	14 (50%)	20 (71.4%)	15 (53.6%)
1	10 (35.7%)	4 (14.3%)	9 (32.1%)
2	4 (14.3%)	2 (7.1%)	2 (7.1%)
3	0	2 (7.1%)	2 (7.1%)
kappa	0.33	0.62	0.4

Detection of individual genoptypes was compared between duplicates. The msp1F3 allelic composition was identical in 12/28 (42.9%) sample pairs, that of MS16 in 19/28 (67.9%).

The probability of detecting a clone in the duplicate samples (n=28) was estimated using the formula presented by Bretscher et al [6] (Table S4).

Table D: Detection of clones in 28 samples genotyped in duplicate

	*msp1*F3	MS16
Clones detected twice	53	61
Clones detected only once	26	16
Detectability [0.95 CI]	0.80 [0.73-0.88]	0.88 [0.0.83-0.94]

The congruence obtained from these 28 samples genotyped in duplicate was compared to detectability of clones. Detectability was estimated from sample pairs collected 24 hours apart from the same child [7]. Fluctuating parasite densities from one day to another lead to differences in bleeds collected within short time intervals, but no gain or loss of infection is expected within such a short time period. When day 1 and day 2 bleeds from the same child were analyzed, detectability was 0.73 for *msp1*F3 clones and 0.61 for MS16 clones [7]. This comparison revealed that the congruence of results obtained from duplicated typing of the same blood sample was much higher, 0.80, for *msp1*F3 and 0.88 for MS16.

Imperfect detectability of parasite clones in field samples is caused by low densities of parasites. Depending on the presence or absence of a parasite clone in the DNA template added to the PCR reaction mix, PCR detection of low-density parasites leads to a chance result [8]. Results obtained at the detection limit of PCR can be exemplified by performing serial dilutions of a DNA solution followed by PCR (see paragraph 3 below).

Conclusion: The agreement of MOI and clone detection in the duplicate samples was reasonable and so genotyping the same sample in duplicate is not necessary.

3. Detection of clones in serial dilutions of DNA

Any method to detect parasites at densities close to the detection limit is imperfect. To assess the impact of low concentrations of template DNA on the outcome of our *P. vivax* genotyping PCR, DNA from field samples was diluted $1{:}10^2$, $1{:}10^3$, $1{:}10^4$ and $1{:}10^5$. Subsequently, *P. vivax* genotyping was performed in triplicate using the standard protocol [7]. Six field samples were analyzed. MOI and allelic composition in each sample were assessed. Results for marker *msp1*F3 are listed in Table S5 and for MS16 in Table S6.

Table E: Number of *msp1*F3 clones detected in triplicates of serial DNA dilutions

DNA dilution	1:1			$1{:}10^2$			$1{:}10^3$			$1{:}10^4$			$1{:}10^5$			Total no. of clones observed
triplicate	A	B	C	A	B	C	A	B	C	A	B	C	A	B	C	
Sample 1*	3	3	3	3	2	2	2	2	1	0	0	0	0	0	0	3
Sample 2	3	4	4	4	3	4	2	1	2	1	0	1	0	0	0	4
Sample 3	2	2	2	1	1	2	0	1	1	1	0	1	0	0	0	2
Sample 4*	3	3	3	3	3	3	2	1	1	0	0	0	0	0	0	3
Sample 5	2	2	2	1	1	2	0	1	0	0	0	0	0	0	0	4
Sample 6	1	3	1	0	2	0	0	0	0	0	0	0	0	0	0	3
Total no. of pos. samples (n=18)	18/18			16/18			12/18			4/18			0/18			

* Electropherogramms are displayed in Figure 1.

Table F: Number of MS16 clones detected in triplicates of serial DNA dilutions.

DNA dilution	1:1			$1{:}10^2$			$1{:}10^3$			$1{:}10^4$			$1{:}10^5$			Total no. of clones observed
triplicate	A	B	C	A	B	C	A	B	C	A	B	C	A	B	C	
Sample 1	3	3	2	2	2	2	2	0	0	0	0	1	0	0	0	3
Sample 2	2	3	3	2	2	3	1	1	3	1	0	0	0	0	0	4
Sample 3	2	2	2	0	2	2	2	0	4	1	0	0	0	0	1	5
Sample 4	4	3	3	4	5	4	3	2	1	1	0	0	0	0	0	5
Sample 5	4	3	4	2	1	1	0	0	0	0	0	0	0	0	0	4
Sample 6	3	1	2	0	0	0	0	0	1	0	0	0	0	0	0	4
Total no. of pos. samples (n=18)	18/18			14/18			10/18			4/18			1/18			

Genotyping results from undiluted and serial dilutions of DNA from field samples 1 and 4, done in triplicate, are shown in Figure 1a and 1b, respectively. At a DNA dilution of 1:10^3, some clones are no longer amplified, as compared to undiluted DNA. At such low DNA concentrations, the distribution of detected genotypes seems to be random, as seen in sample 4 (Figure 1b): one or two genotypes per PCR reaction were amplified at a DNA dilution of 1:10^3, but the allelic composition of the 3 replicates differed. Triplicate A showed two fragments of 236 bp and 262 bp, respectively. Triplicate B contained a single 262 bp fragment only, and triplicate C a single 274 bp fragment. All 3 clones were amplified when DNA was not diluted.

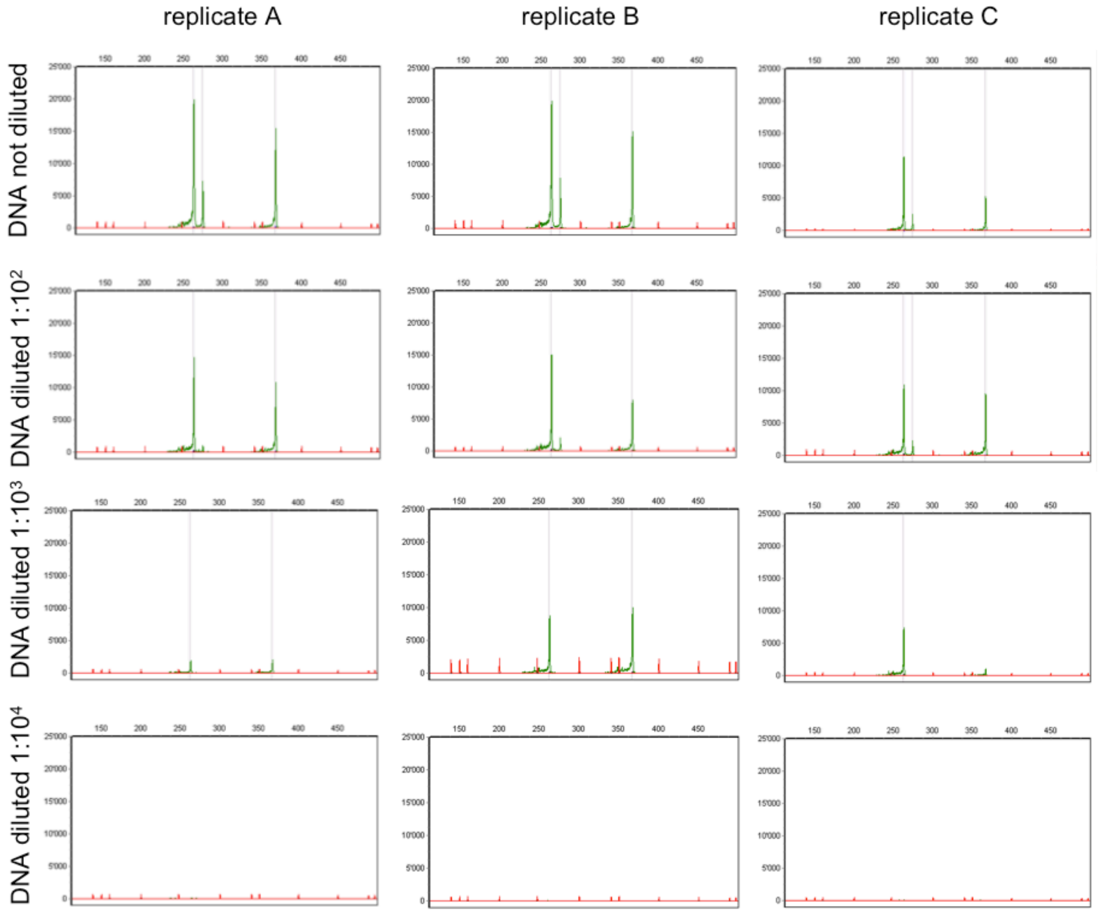

Figure A: Electropherogrammes of *msp1*F3 genotyping PCR performed in triplicate (relicates A, B,and C) on serial dilutions of field sample 1. The standard cut-off of 1000 RFU was applied for all samples. Alleles above this cut-ff are indicated with a grey line

X-axis: size of DNA fragment in base pairs
y-axis: Relative fluorescent units (RFU)
Red: commercial size standard
Green: *msp1*F3 alleles

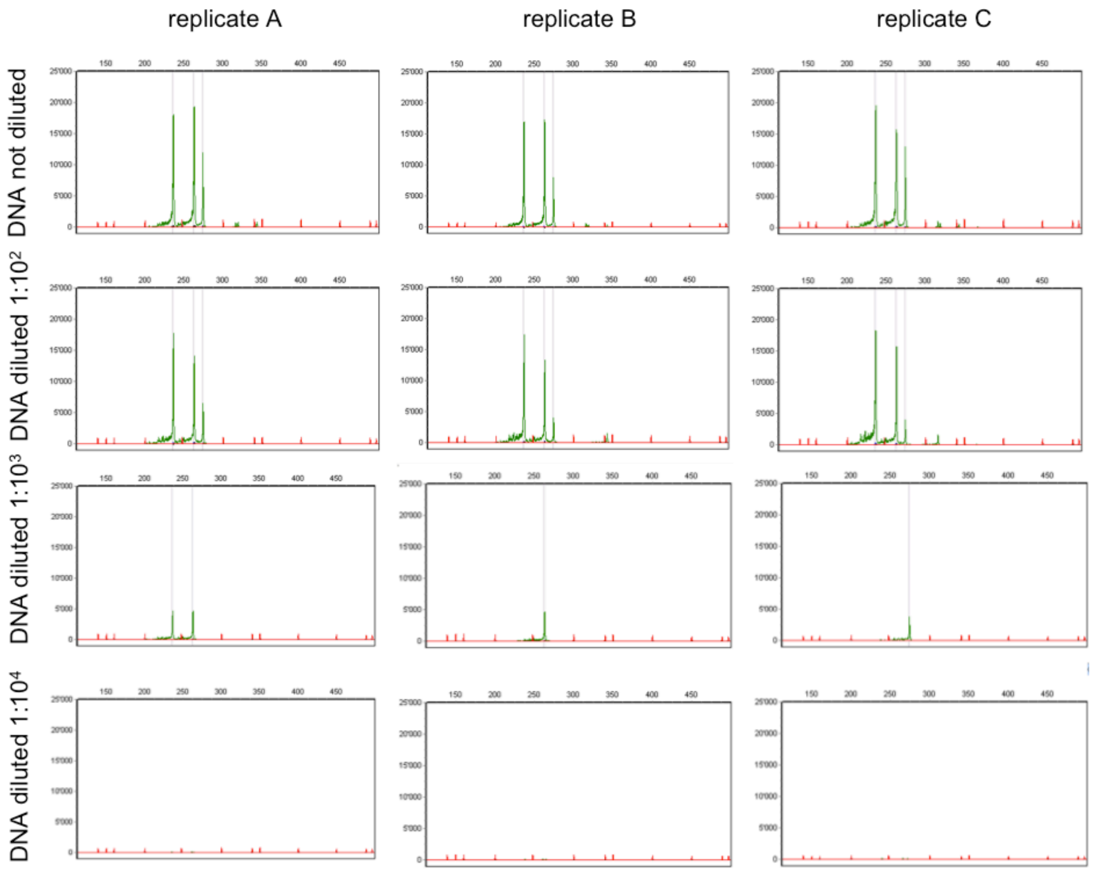

Figure B: Electropherogrammes of *msp1*F3 genotyping PCR performed in triplicate (replicates A, B,and C) on serial dilutions of field sample 4.

Conclusion: When low parasitemia is simulated by serial DNA dilutions, detection of clones becomes stochastic and differs between replicates.

References

1. Karunaweera ND, Ferreira M. U., Hartl D. L., Wirth D. F. (2006) Fourteen polymorphic microsatellite DNA markers for the human malaria parasite *Plasmodium vivax*. Molecular Ecology Notes 7: 172-175.
2. Brito CFAd, Ferreira MU (2011) Molecular markers and genetic diversity of Plasmodium vivax. Mem Inst Oswaldo Cruz 106.
3. Havryliuk T, Ferreira MU (2009) A closer look at multiple-clone Plasmodium vivax infections: detection methods, prevalence and consequences. Mem Inst Oswaldo Cruz 104: 67-73.
4. Orjuela-Sanchez P, da Silva NS, da Silva-Nunes M, Ferreira MU (2009) Recurrent parasitemias and population dynamics of Plasmodium vivax polymorphisms in rural Amazonia. Am J Trop Med Hyg 81: 961-968.
5. Imwong M, Nair S, Pukrittayakamee S, Sudimack D, Williams JT, et al. (2007) Contrasting genetic structure in *Plasmodium vivax* populations from Asia and South America. Int J Parasitol 37: 1013-1022.
6. Bretscher MT, Valsangiacomo F, Owusu-Agyei S, Penny MA, Felger I, et al. (2010) Detectability of Plasmodium falciparum clones. Malar J 9: 234.
7. Koepfli C, Schoepflin S, Bretscher M, Lin E, Kiniboro B, et al. (2011) How much remains undetected? Probability of molecular detection of human plasmodia in the field. PLoS ONE 6: e19010.
8. Ishengoma DS, Lwitiho S, Madebe RA, Nyagonde N, Persson O, et al. (2011) Using rapid diagnostic tests as source of malaria parasite DNA for molecular analyses in the era of declining malaria prevalence. Malar J 10: 6.
