# Supplementary material for: Multiplicity and Diversity of Plasmodium vivax Infections in a Highly Endemic Region in Papua New Guinea
Source: PLoS Negl Trop Dis. 2011 Dec 20;5(12):e1424. doi: 10.1371/journal.pntd.0001424 (PMC3243695; doi:10.1371/journal.pntd.0001424)
Supplement: Checklist S1 — STROBE checklist. (DOC) [file pntd.0001424.s003.doc]

STROBE Statement—Checklist of items that should be included in reports of ***cohort studies***

Citation: Koepfli C et al. Multiplicity and diversity of *Plasmodium vivax* infections in a highly endemic region in Papua New Guinea. PLoS Negl Trop Dis

|  | Item No | Recommendation |
| --- | --- | --- |
| **Title and abstract** | 1 | (*a*) Indicate the study’s design with a commonly used term in the title or the abstract  Abstract |
|  |  | (*b*) Provide in the abstract an informative and balanced summary of what was done and what was found  Given in Abstract |
| Introduction | | |
| Background/rationale | 2 | Explain the scientific background and rationale for the investigation being reported  Introduction, Paragraph 2 |
| Objectives | 3 | State specific objectives, including any prespecified hypotheses  Introduction, Paragraph 3 |
| Methods | | |
| Study design | 4 | Present key elements of study design early in the paper  Methods, Paragraph 2+3 |
| Setting | 5 | Describe the setting, locations, and relevant dates, including periods of recruitment, exposure, follow-up, and data collection  Methods, Paragraph 2+3, reference 8 |
| Participants | 6 | (*a*) Give the eligibility criteria, and the sources and methods of selection of participants. Describe methods of follow-up  (*a*) Methods, Paragraph 3, reference 8 |
|  |  | (*b*) For matched studies, give matching criteria and number of exposed and unexposed Not applicable |
| Variables | 7 | Clearly define all outcomes, exposures, predictors, potential confounders, and effect modifiers. Give diagnostic criteria, if applicable  Methods, Paragraph 3 (diagnostic criteria), reference 8 |
| Data sources/ measurement | 8* | For each variable of interest, give sources of data and details of methods of assessment (measurement). Describe comparability of assessment methods if there is more than one group Methods, Section “Data Analysis” |
| Bias | 9 | Describe any efforts to address potential sources of bias Reference 8 |
| Study size | 10 | Explain how the study size was arrived at Reference 8 |
| Quantitative variables | 11 | Explain how quantitative variables were handled in the analyses. If applicable, describe which groupings were chosen and why Methods, Section “Data Analysis”, paragraph 3 |
| Statistical methods | 12 | (*a*) Describe all statistical methods, including those used to control for confounding Methods, Section “Data Analysis” |
|  |  | (*b*) Describe any methods used to examine subgroups and interactions Methods, Section “Data Analysis”, paragraph 3 |
|  |  | (*c*) Explain how missing data were addressed Methods, section “Species detection, genotyping and data analysis”, paragraph 6 |
|  |  | (*d*) If applicable, explain how loss to follow-up was addressed Reference 8 |
|  |  | (*e*) Describe any sensitivity analyses Not applicable |
| Results | | |
| Participants | 13* | (a) Report numbers of individuals at each stage of study—eg numbers potentially eligible, examined for eligibility, confirmed eligible, included in the study, completing follow-up, and analysed Results, paragraphs 1+2 |
|  |  | (b) Give reasons for non-participation at each stage Reference 8 |
|  |  | (c) Consider use of a flow diagram  Not necessary |
| Descriptive data | 14* | (a) Give characteristics of study participants (eg demographic, clinical, social) and information on exposures and potential confounders Methods, Paragraph 3, reference 8 |
|  |  | (b) Indicate number of participants with missing data for each variable of interest Reference 8 |
|  |  | (c) Summarise follow-up time (eg, average and total amount) Reference 8 |
| Outcome data | 15* | Report numbers of outcome events or summary measures over time Results, paragraphs 1+2 |
| Main results | 16 | (*a*) Give unadjusted estimates and, if applicable, confounder-adjusted estimates and their precision (eg, 95% confidence interval). Make clear which confounders were adjusted for and why they were included  Results, Methods section (confounders adjusted) |
|  |  | (*b*) Report category boundaries when continuous variables were categorized Methods, Data Analysis, paragraph 3 (age groups) |
|  |  | (*c*) If relevant, consider translating estimates of relative risk into absolute risk for a meaningful time period No risk indicators analyzed |
| Other analyses | 17 | Report other analyses done—eg analyses of subgroups and interactions, and sensitivity analyses Not applicable |
| Discussion | | |
| Key results | 18 | Summarise key results with reference to study objectives Discussion, paragraph 2 |
| Limitations | 19 | Discuss limitations of the study, taking into account sources of potential bias or imprecision. Discuss both direction and magnitude of any potential bias Discussion, paragraph 7 |
| Interpretation | 20 | Give a cautious overall interpretation of results considering objectives, limitations, multiplicity of analyses, results from similar studies, and other relevant evidence All Discussion |
| Generalisability | 21 | Discuss the generalisability (external validity) of the study results Discussion, paragraph 5 |
| Other information | | |
| Funding | 22 | Give the source of funding and the role of the funders for the present study and, if applicable, for the original study on which the present article is based Submitted separate to manuscript on PLoS NTDs submission page |

*Give information separately for exposed and unexposed groups.

**Note:** An Explanation and Elaboration article discusses each checklist item and gives methodological background and published examples of transparent reporting. The STROBE checklist is best used in conjunction with this article (freely available on the Web sites of PLoS Medicine at http://www.plosmedicine.org/, Annals of Internal Medicine at http://www.annals.org/, and Epidemiology at http://www.epidem.com/). Information on the STROBE Initiative is available at http://www.strobe-statement.org.
